# Evidence Generation of Standard Nursing Protocol on Chemotherapy-Induced Neutropenia Among Oncology Nurses

**DOI:** 10.7759/cureus.31217

**Published:** 2022-11-07

**Authors:** Payal R Bawankar, Ruchira Ankar

**Affiliations:** 1 Nursing, Smt. Radhikabai Memorial College of Nursing, Datta Meghe Institute of Medical Sciences (Deemed to be university), Wardha, IND

**Keywords:** oncology nurses, chemotherapy-induced neutropenia, practice, knowledge, standard nursing protocol

## Abstract

Background

Chemotherapy uses anti-neoplastic agents, the drugs used to treat malignancies. Neutropenia is associated with cytotoxic therapy. Anti-neoplastic agents are poisonous to the cells, affecting the synthesis of folic acid and damaging the DNA, RNA, and protein that damage the bone marrow. Destruction of bone marrow decreases absolute neutrophil count in the blood. To assess the baseline data and practice of oncology nurses, develop and implement the evidence-based standard nursing protocol, assess the satisfaction level of the patients for the utility of the standard nursing protocol, find out the correlation between knowledge and practice and to associate the knowledge and practice score with selected demographic variables.

Material And Method

In the study quasi-experimental design was utilized. The study was conducted at Siddharth Gupta Memorial Cancer Hospital, Sawangi (Meghe), Wardha city, between June to Aug 2022

Result

The result of the study shows the mean value per existing knowledge is 7.59 and practices is 37.95, post knowledge is 14.23, and post practices is 71.15. The standard deviation values of per exiting knowledge are 2.67 and, practice 3.09 and, post knowledge is 2.32, post practices are 1.32. The calculated t-value of knowledge is 46.57and the p-value is 0001. The calculated t-value of practice is 12.03, and the p-value is 0.0001.

Conclusion

The present research concluded that the knowledge and skills of oncology nurses are enhanced after implementing the standard nursing protocol.

## Introduction

Cardiovascular diseases (CVD) are the leading cause of mortality, accounting for 73% of deaths worldwide [1,2]. Cancer is the second leading cause of mortality, accounting for 10 million deaths worldwide [3,4]. Chemotherapy uses anti-neoplastic agents to treat different malignancies. The goal of cytotoxic therapy is to decrease the number of malignant cells in cancer, such as leukemia, or to reduce the size of tumors and thereby lessen the severity of the symptoms. Anti-neoplastic agents inhibit cell replication by interfering with the specific cell phase, inhibiting the enzymes, or damaging the target cell's DNA, RNA, and protein synthesis, which leads to bone marrow destruction and depletion in neutrophil production [5]. Neutropenia occurs due to the depletion of the absolute neutrophil count in a cancer patient's blood. Neutropenia occurs in 1 in 3 cancer patients treated with cytotoxic therapy. Anti-neoplastic drugs schedule and dose to be delayed sometimes to give the body a chance to produce new neutrophils [6].

In neutropenia, absolute neutrophil count decrease <500 cells/mm3 in the next 48hours. Body circulating white blood cells consist of 60% of neutrophils. Mostly WBC reached their lowest point 7-14 days after the administration of chemotherapy [7]. Oncology nurses play a crucial role in preventing, early diagnosing, and managing CIN neutropenia and educating patients and their families to ensure better compliance rates. Oncology nurses must detect the patient at risk of developing neutropenia early and monitor them for better implementation of nursing interventions to improve the care and quality of life of patients undergoing CIN [8]. Nurses must require specialized skills and knowledge beyond basic nursing to understand the psychological, physical, and cost implications of supporting the patient and family integrity during the CIN experience [9].

The development of a standard nursing protocol should be based on oncology care guidelines and need-based assessment of nurses, patients, and families giving care to the patients. The standard nursing protocol can guide nurses and patient caregivers in making decisions for adequate care for cancer patients. Therefore, it is essential to develop and implement standard nursing protocols to reduce infection and improve care quality. Evidence-based practices are expanding rapidly in support of nursing intervention, generating a demand to bring this new education and specialized skills to the bedside [10]. To identify evidence and their base for the oncology nurses and illustrate the impact of nursing interventions on patient outcomes, the oncology nursing society is committed to managing specific cancer symptoms [11]. The evidence-based practice consists of the conscious application of various skills and knowledge sources, including published articles on clinical practices, patient value, and care according to the priority of the patient [12]. 

## Materials and methods

Study setting and design

 A quasi-experimental research design was used in the current study. The study was conducted at Siddharth Gupta Memorial Cancer Hospital, Sawangi (Meghe), Wardha city, between June to Aug 2022. Oncology nurses working in the oncology ward were selected for this research study

Study population

Non-probability purposive sampling technique was utilized in this study. A total of 100 oncology nurses were selected for the study, in addition to 50 cancer patients who are on chemotherapy.

Study tools

The tool consists of four sections: tool I was generated to collect data on oncology nurses' age and years of experience; tool II, staff nurses' pre-test/post-test questionnaire, was used to measure oncology nurses' knowledge. This tool was given to nurses before the implementation of the SNP and after seven days to assess the gain in knowledge and change in practice after the implementation of the protocol, and it consists of 20 knowledge questions; tool III consists of the observation checklist data from this tool were collected before discussion of the protocol and after seven days to evaluate the impact of the SNP on oncology nurses' practice. It consisted of the following 24 items, including neutropenia stratification, calculation of absolute, assessment and monitoring of cancer patients on chemotherapy and nursing intervention to reduce infection; tool IV consists of patient feedback from data from these tools were collected from patients after the discussion of a protocol to evaluate the impact of the standard nursing protocol in reduction of infection and to know nurses practicing the protocol is effective in decreasing infection.

The tool was submitted to 3 medical oncologists and seven nursing experts to ensure content validity. Reliability analysis was done using the parallel form reliability method, which is found to be 0.94; the tool is reliable. The pilot study was done in Jan 2022 per the criteria for 10 samples collected from the Rast. Sant Tukdoji cancer hospital, Nagpur.

Data collection

Data collection was done in four steps; in the first step, staff nurses gave a knowledge questionnaire and neutropenic patient management observation checklist, which contained 24 items to evaluate the oncology nurses' practice. They were asked to fill in some socio-demographic data like age and years of experience. Oncology nurses' knowledge and practice were measured to be utilized as baseline data before the nursing protocol implementation. On the same day, the standard nursing protocol was given to the staff nurses in the form of a booklet and demonstrated and re-demonstrated with the main teaching methods. This was done in 5 sessions for each group of 10-20 nurses, and the duration was 30-60 min. In the second step, a post-test was collected from staff nurses after seven days. In the third step, feedback is taken from the staff nurses, and in the fourth step, data collected is entered in the Excel sheet.

Statistical analysis

Data collected were analyzed using the software SPSS statistics version 24.0. Using descriptive statistics (Socio-demographic variables were described/expressed in frequencies and percentages, mean percentage, mean and standard deviation) and inferential statistics "paired t-test" was used to assess the effectiveness of the standard nursing protocol. Item analysis was done on an observation checklist, and the correlation coefficient was used to find the correlation between knowledge and expressed practices. The chi-square test was used to determine the association of knowledge and expressed practice with demographic data. The acceptable level of significance is NS,p>0.05.

Ethical consideration

Informed consent was taken from each staff nurse and patient after a detailed explanation of the study. The participants were ensured privacy and confidentiality. Permission is obtained from the concerned authority of the selected hospital. The study proposal has been sanctioned by the ethical committee of the college (referral no: DMIMS(DU)/IEC/2021/282).

## Results

A convenient sample of 100 nurses was selected based on the distribution of oncology nurses about their demographic characteristics. About 40% were in the 31-40 years, 35% were in 21-30 years, 18% were in the age group of 41-50 years, and 7% of staff nurses were in the age group of 51-60 years, respectively. 47% of staff nurses had working experience of up to 10 years, 42% had between 11-20 years, 6% had working experience of 21-30 years, and 5% of staff nurses had working experience of 31-40years (Table 1).

**Table 1:** Distribution of oncology nurses according to their demographic characteristics.

Demographic Variables	Frequency (n=100)	Percentage (%)
Age (yr)		
21-30 year	35	35%
31-40 year	40	40%
41-50 year	18	18%
51- 60 year	7	7%
Experience in yrs		
≤ 10 years	47	47%
11- 20 years	42	42%
21- 30 years	6	6%
31- 40 years	5	5%

47% of the oncology nurses had an average score, 32% had a poor one, 16% had a good, and 5% of oncology nurses had an excellent knowledge score. The mean score was 7.59±3.97, and the mean percentage was 37.95±19.87 (Table 2).

**Table 2:** Assessment of pre-test knowledge score

Knowledge Score	Score Range	Pre-test	
		Frequency	Percentage
Poor	0-25% (1-5)	32	32%
Average	26-50% (6-10)	47	47%
Good	51-75% (11-15)	16	16%
Excellent	76-100% (16-20)	5	5%
Minimum		2	
Maximum		18	
Mean		7.59±3.97	
Mean%		37.95±19.87	

In the post-test, 64% of oncology nurses scored well, and 35% had excellent knowledge. The minimum score in the post-test was 10, and the maximum was 19. The mean was 14.23±2.50, and the mean percentage obtained was 71.15±12.50 (Table 3).

**Table 3:** Assessment of post-test knowledge score

Knowledge Score	Score Range	Post-test	
		Frequency	Percentage
Poor	0 – 25% (1-5)	0	0
Average	26 – 50% (6-10)	1	1%
Good	51- 75% (11-15)	64	64%
Excellent	76-100% (16-20)	35	35%
Minimum		10	
Maximum		19	
Mean		14.23± 2.50	
Mean%		71.15 ± 12.50	

In the pre-test, 46% of staff nurses had average, 24% had poor, 20% had good, and 10% had excellent practice scores. The minimum practice score was three, and the maximum was 23. The mean was 10.69±5.35, and the mean percentage was 44.54±22.30 (Table 4).

**Table 4:** Assessment of pre-test practice score

Practice Score	Score Range	Pre-test	
		Frequency	Percentage
Poor	0-6	24	24%
Average	7-12	46	46%
Good	13-18	20	20%
Excellent	19-24	10	10%
Minimum		3	
Maximum		23	
Mean		10.69 ± 5.35	
Mean%		44.54 ± 22.30	

In the post-test, 41% of oncology nurses had a good, 59% had an excellent, and 41% had a good practice score. The minimum practice score was 13, and the maximum was 24. The mean score was 18.44±2.94, and the mean percentage was 76.83±12.28 (Table 5).

**Table 5:** Assessment of post-test practice score

Practice Score	Score Range	Post - test	
		Frequency	Percentage
Poor	0-6	0	0
Average	7-12	0	0
Good	13-18	41	41%
Excellent	19-24	59	59%
Minimum		13	
Maximum		24	
Mean		18.44 ± 2.94	
Mean%		17.83 ± 12.28	

The comparison between pre-test and post-test knowledge and practice scores of oncology nurses. The calculated 't' value, i.e., 14.98, is much higher than the tabulated value at a 5% significance level for the overall knowledge, which is a statistically acceptable significance level. The tabulated value for n=100-1, i.e., 99 degrees of freedom, was 1.98. The estimated 't' value, i.e., 12.03, is much higher than the calculated value at a 5% significance level for the overall practice score, which is a statistically acceptable significance level. Hence it is statistically interpreted that the nursing protocol regarding CIN among oncology nurses was effective. Thus, the H1 is accepted (Table 6).

**Table 6:** Association of pre and post-test knowledge and practice score

Paired t-test	Overall	Mean	SD	Mean difference	t - value	P - value
Knowledge	Pre test	7.59	3.97	6.64±4.43	14.98	0.0001 S,p<0.05
	Post test	14.23	2.50			
Practice	Pre- test	10.69	5.35	7.75±6.43	12.03	0.0001 S,p<0.05
	Post- test	18.44	2.94			

The nurses give care to the patient appropriately as feedback collected from the patient shows that nursing care is effective and fever is reduced. Irritation is reduced after catheter care, and they follow the standard nursing protocol, which helps them reduce chemotherapy-induced neutropenia (Table 7).

**Table 7:** Feedback analysis

Sr no.	Nursing action	agree	disagree
1	Detailed history regarding chemotherapy-induced neutropenia taken	50(100%)	0(0%)
2	Physical examination done properly	50(100%)	0(0%)
3	Temperature, blood pressure, oxygen saturation checked properly and management of fever and blood pressure done properly	50(100%)	0(0%)
4	All investigation done	50(100%)	0(0%)
5	Before and after doing the procedure nurses do handwashing or use sanitizer	47(94%)	3(6%)
6	Oral care is given thrice a day and helps you to prevent mouth ulcers and mouth odor	48(96%)	2(4%)
7	Intravenous line dressing is changed every 4 hourlies	47(94%)	3(6%)
8	Proper Indwelling catheter care is provided which helps you to prevent irritation	47(94%)	3(6%)
9	Nurses wear masks and gloves before giving care	45(90%)	5(10%)
10	Fever and infection reduced after giving care	41(82%)	9(18%)
11	Restricted animals near them because they may cause infection	41(82%)	9(18%)
12	Avoided keeping fresh or dried flowers and plants in the room	37(74%)	13(23%)
13	The avoided crowd only one or two visitors should allow for meeting at a time	50(100%)	0(0%)
14	Given information regarding the prevention of infection you and your relative	49(99%)	1(2%)
15	Avoid contact with individuals suffering from respiratory disease	47(94%)	3(6%)

The correlation between post-test knowledge and practice scores of oncology nurses. The correlation was done with the help of Pearson's correlation coefficient, and students' unpaired 't-test was applied at a 5% significance level. Hence it is statistically interpreted that a positive correlation was established between knowledge and practice scores (Table 8).

**Table 8:** Correlation between knowledge score and Practice score

Overall	Mean	SD	Mean difference	r - value	P - value
Knowledge Score	14.23	2.50	4.21± 3.47	0.19	0.054 NS, p> 0.05
Practice Score	18.44	2.94			

The association of practice scores with age and experience in years of staff nurses. It is interpreted that the age and experience of staff nurses are statistically associated with their post-test practice scores (Table 9).

**Table 9:** Association of the post-test and pretest practice score

Demographic data		Frequency		Mean practice score	F - value	P - value
Age	21-30 yrs	35		19.34±2.87	3.74	0.014, S,p<0.05
	31-40 yrs	40		17.30±3		
	41-50 yrs	18		18.83±2.81		
	51-60 yrs	7		19.42±0.53		
Experience	≤10 yrs		47	19.53±2.70	12.49	0.0001 S,p<0.05
	11-20 yrs		42	16.88±2.81		
	21-30 yrs		6	19.72±1.48		
	31-40 yrs		5	20.21±1.62		

## Discussion

The present study aims to generate an evidence-based standard nursing protocol on CIN for oncology nurses. The study findings reveal that oncology nurses have insufficient knowledge and unsatisfactory skills before the implementation of standard nursing protocols after. This reflects that nurses require proper training, in-service program, and guidelines for caring for patients with a high risk of developing CIN.

The study is supported by a descriptive cross-sectional study conducted to evaluate nursing students' knowledge about the management of CIN. The sample selected for the study is 230 nursing students from the nursing colleges of Saudi Arabia. The study finding reveals that nursing students have a poor knowledge mean of 10.1. The bridging students had higher Score M = 12.6 & SD = 9.8 the regular students had M = 9.8 &SD = 5.5, [t = 2.9, df = 38.9, p = 0.006]. Students who had previous training about Neutropenia [M = 11.6, SD = 5.0] had higher knowledge than the students who did not get any training previously [M = 9.5, SD= 5.6][t= -2.73, df= 134, p= 0.007]. The study concluded that it is essential to improve nurses' quality of care and knowledge regarding neutropenia management [13].

A descriptive study supports the study to assess the knowledge of preventing infections in patients undergoing chemotherapy among student nurses. Non-probability purposive sampling technique was used, and 60 samples were selected for data collection. 50% of subjects were in the age group of below 20 years, and females were 95%. Among 60 students 40(66.7%) had adequate knowledge 20(33.3%) had inadequate knowledge. The mean score of knowledge is 40.90%. The study's finding reveals an association between the knowledge of student nurses and selected demographic variables exposed to mass media [14].

A cross-sectional survey supports the study to evaluate nurses' knowledge of CIN. The finding of study participants aged 34 years (78%) was female and with an undergraduate degree in nursing (95.6%). The nurses had an average knowledge mean total score is 16.4. The nurses had a post-graduation degree P = 0.048, had attended a training P = 0.011, had completed an education course on Neutropenia P= 0.07, were working in an oncology unit P = 0.002, and had to experience an oncology hospital P= 001, were having more knowledge of CIN as compare to others. The study concluded that plans for training and in-service education on CIN are other choices with a more impact on the knowledge and practice of nurses [15].

The Present study is supported by the methodological study utilized to develop a protocol for nurses and caregivers related to the prevention, early detection, and management of CIN. After analysis of different studies and review and assessing the practice of nurses and families, the first draft was prepared. Three Delphi rounds were conducted to validate the protocol by experts so that the protocol in the form of a booklet is feasible. A checklist also develops, which was used to evaluate the protocol. The protocol and checklist Content Validity Index (CVI) was 100%. Overall, Cronbach's alpha value was 0.859. It is concluded that the study has provided valid and reliable written guidelines for nurses and caregivers concerning the prevention, early detection, and management of CIN complications [16].

Limitations of the study

Our study has a few limitations. Primarily, our sample size (100) was small, and the patient outcome and follow-up were not evaluated in this study.

Strengths of the study

Although the study was a small-scale analysis due to the availability of fewer oncology nurses in one hospital and time limitation, it showed that standard nursing protocols are important interventions that would help to increase the level of knowledge and practice among staff nurses to reduce the neutropenia, especially in the field of oncology. We also have taken the feedback, which helps us to generate evidence that nurses implementing the protocol and it helps reduce infection.

## Conclusions

Designing and executing evidence-based standard nursing procedures is crucial for enhancing patient satisfaction and nurses' knowledge and practice. By providing education, information, and assessment while influencing rules and procedures, nurses can proactively manage CIN. The results of the current study confirmed this. After introducing the protocol, nurses' skills and knowledge for managing CIN patients after chemotherapy have greatly improved.

## Appendices

Chemotherapy-induced neutropenia (CIN)

Standard Nursing Protocol (SNP)

Standard deviation (SD)

                                                         STRUCTURED QUESTIONNAIRE

Sample No………

INSTRUCTION

Read all questions carefully.

Select the only alternative which is most appropriate and tick mark (√) against it .

SECTION A:

Demographic data

1. Age

--------------

2. Work experience in years

------------------------

SECTION B

Choose the appropriate answer and put a (√) mark against the option 

1. In chemotherapy-induced neutropenia depletion of

a)     White blood cells

b)     Red blood cells

c)     Granulocytes

d)     Platelets

2. Neutrophils are synthesized and produced by

a)     Mesenchymal stem cell

b)     Neural stem cell

c)     Hematopoietic stem cell

d)     Epithelial stem cell

3. In mild neutropenia absolute neutrophil count is

a)     >1.0 x 109

b)          1.0-1.5 x 109

c)     0.5-1.0 x109

d)     < 0.5 x 109

4. Neutropenia called as severe if it exceeds from

a)     8 to 9 days

b)     11 to 13 days

c)     10 to 15 days

d)     10 to 14 days

5. In cell-cycle-specific drugs nadir occur in

a)     7 to 14 days

b)     10 to 15 days

c)     7 to 12 days

d)     5 to 14 days

6. In children due to some cell cycle nonspecific drugs nadir occurs in

a)     21 to 35 days

b)     23 to 30 days

c)     20 to 30 days

d)     15 to 32 days

7. Disease-specific risk factors of neutropenia are

a)     Pneumonia

b)     Lung cancer

c)     Chemotherapy regimen

d)     Hypertension

8.    MASCC score of ≥ 21 is considered 

a)     High risk

b)     No risk

c)     Mild risk

d)     low risk

9. During high-risk assessment what do we have to assess

a)     Outpatient status at the time of development of fever

b)     No concomitant acute comorbid illness

c)     Hepatic or renal insufficiency

d)     Pneumonia or other complex infections at clinical presentation

10. During history collection review of current antibiotic regimens reveals the use of agents such as

a)     Amphotericin B

b)     Cephalexin

c)     Ciprofloxacin

d)     Sulfamethoxazole

11. In neutropenic patient during skin examination abnormalities is present

a)     Contact dermatitis

b)     Hives

c)     Psoriasis

d)     Erythema

12. In neutropenic patients during throat examination oropharynx may reveal

a)     Laryngitis

b)     Tonsillitis

c)     Sore throat

d)     Ulcers

13. In neutropenic patients chest x-ray reveals

a)     Diffuse infiltrates

b)     Heart-related lung problems

c)     Calcium deposits

d)     Blood vessels

14.  Lactate test is done in neutropenic patients to diagnose

a)     Lactic acidosis

b)     Metabolic acidosis

c)     Metabolic alkalosis

d)     Lactic alkalosis

15. Medical handwashing should be done till

a)     15sec

b)     12sec

c)     10sec

d)     1min

16. Neutropenic patient oral care includes rinsing of the mouth at least

a)     4 times a day

b)     5 times a day

c)     3 times a day

d)     2 times a day

17. Examination of the cannulation site is necessary to know

a)     Inflammation

b)     Swelling

c)     Phlebitis

d)     All of the above

18. Neutropenic patients should avoid crowding gatherings because

a)     It helps to maintain silence

b)     It can prevent infection

c)     It can prevent irritation

d)     It helps for better treatment

19. If fresh and dried flowers are kept inpatient room can cause a risk of

a)     Aspergillus infection

b)     Blastomycosis

c)     Neoformans infection

d)     Coccidioidomycosis

20. Portable ultrasound bladder scans are useful to detect

a)     Renal calculi

b)     Nephritis

c)     residuals urine amounts

d)     Urinary tract infection

Answer key of 20 knowledge questionnaires (Table 10).

**Table 10:** ANSWER KEY

QUESTION NO	ANSWER KEY	QUESTION NO	ANSWER KEY
1	A	11	D
2	C	12	D
3	B	13	A
4	D	14	D
5	A	15	A
6	A	16	C
7	B	17	C
8	D	18	B
9	C	19	A
10	A	20	C

Tool II:- observation checklist consists of 24 items to evaluate the practice of oncology nurses (Table 11). Tool III:- Feedback form which shows that nursing care is effective and infection is reduce  (Table 12).                                          

**Table 11:** Observation checklist [17].

Sr no.	Content	yes	No
	Calculate the absolute neutrophil count		
	Determine the risk and duration of neutropenia		
	Identify the nadir		
	Identify the risk factor for neutropenia and related events		
	Calculate the mask index score for the adults		
	Assessed the neutropenic patient who are at “low-risk” and “high-risk” for medical complications.		
	Checked ECOG performance status of patient		
	Document history of neutropenic patient		
	Duration of the symptoms		
	A description of the onset of symptoms		
	Anatomic location of the symptoms		
	Severity of the symptoms		
	Alleviating and aggravating factors		
	Previous history of similar symptoms		
	Initial clinical assessment check		
	Temperature		
	Pulse		
	Blood pressure		
	Respiration rate		
	Oxygen saturation		
	Fluid balance (input and output charting)		
	Initial Investigations		
	Biochemical profile		
	C-reactive protein Full blood count		
	Peripheral blood cultures (irrespective of temperature)		
	Blood cultures from venous access device including each lumen if applicable		
	Serum lactic acid (if signs of ongoing clinical deterioration		
	Before and after every procedure handwashing is done		
	Proper handwashing should be done till 15min		
	After handwashing with alcohol-based solution dried hand till 20-30sec		
	Oral care given thrice a day and if needed		
	Asses the intravenous line for patency, erythema, tenderness, pain, swelling, dressing integrity and position		
	Intravenous line dressing changed every 4 hourly		
	Cleaned the skin around the IV line with chlorhexidine solution		
	Uses 0.9 % normal saline for a flush of the intravenous line after every drug administration		
	Written date of IV Cannulation		
	Proper Indwelling catheter care provided to patient		
	Appropriately secure the catheters		
	Maintained good hygiene at the catheter-urethral interface		
	Remove catheters when no longer needed		

**Table 12:** Feedback form [18] .

Sr no.	Nursing action	agree	disagree
1	Detailed history regarding chemotherapy-induced neutropenia taken		
2	Physical examination did properly		
3	Temperature, blood pressure, oxygen saturation checked properly and management of fever and blood pressure done properly		
4	All investigation done		
5	Before and after doing the procedure nurses do handwashing or use sanitizer		
6	Oral care is given thrice a day and helps you to prevent mouth ulcers and mouth odor		
7	Intravenous line dressing is changed every 4 hourlies		
8	Proper Indwelling catheter care is provided which helps you to prevent irritation		
9	Nurses wear masks and gloves before giving care		
10	Fever and infection reduced after giving care		
11	Restricted animals near them because they may cause infection		
12	Avoided keeping fresh or dried flowers and plants in the room		
13	The avoided crowd only one or two visitors should allow for meeting at a time		
14	Given information regarding the prevention of infection you and your relative		
15	Avoid contact with individuals suffering from respiratory disease		
